# Reinventing amlodipine

**DOI:** 10.1016/j.jpet.2026.104900

**Published:** 2026-04-24

**Authors:** Bartosz Grzymala, Dagmar Þöll Halldórsdóttir, Haraldur Þorsteinsson, Kristín Þorfinnsdóttir, Hildur Sóley Sveinsdóttir, Matthew O. Parker, Sebastien Foulquier, Karl Ægir Karlsson

**Affiliations:** 1 3Z, Reykjavik, Iceland; 2 Department of Engineering and Natural Sciences, University of Iceland, Reykjavik, Iceland; 3 Surrey Sleep Research Centre, School of Biosciences, University of Surrey, Guildford, Surrey, United Kingdom; 4 Department of Pharmacology and Toxicology, Maastricht University, Maastricht, Netherlands; 5 Biomedical Center, University of Iceland, Reykjavik, Iceland; 6 School of Science and Engineering, Reykjavik University, Reykjavik, Iceland

**Keywords:** Attention-deficit/hyperactivity disorder, l-type calcium channels, Amlodipine, S-amlodipine, Dopamine, Drug repurposing

## Abstract

Attention-deficit/hyperactivity disorder (ADHD) is a prevalent neurodevelopmental disorder, and pharmacological treatments have limited mechanistic specificity. Most nonstimulants target noradrenergic tone but show modest efficacy. l-Type calcium channels (LTCCs) modulate neuronal excitability, catecholaminergic transmission, cortical plasticity, and neuroinflammation, processes central to ADHD pathophysiology. This review evaluates the evidence for repurposing the LTCC blocker amlodipine as a novel ADHD therapeutic. We propose a mechanistic framework where amlodipine acts within attention/arousal circuits to stabilize dopaminergic and noradrenergic tone, restore D2-autoreceptor feedback, enhance plasticity, and reduce neuroinflammation. This systems-level model provides explanatory links between drug action and ADHD pathophysiology, highlighting therapeutic avenues not addressed by current treatments. Contrary to historical assumptions, recent evidence confirms that amlodipine penetrates the blood-brain barrier. Convergent preclinical findings show phenotype rescue in zebrafish and rat models of ADHD, accompanied by normalization of ADHD-relevant metabolic pathways. Complementary biobank analyses suggest reduced traits associated with ADHD among genetically at-risk individuals taking amlodipine. Given enantiomeric pharmacology, *S*-amlodipine, with higher LTCC affinity and fewer off-target liabilities than *R*-amlodipine, emerges as a preferred candidate. Together, these findings warrant a reappraisal of LTCC modulation in ADHD. By providing mechanistic explanations of how amlodipine engages ADHD-relevant circuits, this review clarifies its therapeutic potential and reshapes our understanding of the drug itself, with implications for repurposing, enantiomer-specific development, and broader clinical translation. We outline next steps, including comparative *S*- versus *R*-amlodipine studies, mechanistic dissection of LTCC subtypes in attentional networks, and controlled clinical testing to evaluate amlodipine’s viability as a nonstimulant therapeutic.

**Significance Statement:**

Genetic, preclinical, and translational evidence implicates dysregulated l-type calcium channel signaling in attention-deficit/hyperactivity disorder, providing a mechanistic rationale for evaluating amlodipine as a therapeutic candidate. *S*-amlodipine is the rational development form, as it mediates the principal l-type calcium channel activity, whereas *R*-amlodipine may contribute to nonbeneficial brain or tolerability effects. This review defines a clear translational path focused on *S*-amlodipine, warranting prospective, dose-optimized clinical trials to determine efficacy on attentional and executive dysfunction with acceptable safety.

## 1. Introduction

Attention-deficit/hyperactivity disorder (ADHD) is a prevalent, heterogeneous neurodevelopmental disorder characterized by inattention, hyperactivity, and impulsivity.^1^ It affects ∼5%–7% of children and often persists into adulthood, causing functional impairment and comorbidity across the lifespan.^2^, ^3^, ^4^ Current pharmacotherapy relies on stimulants and nonstimulants, which provides symptomatic relief for many patients but carries significant drawbacks. Stimulants often cause decreased appetite, headaches, hypertension, and sleep disturbances and pose a high abuse risk.^5^ Nonstimulants show lower efficacy and often cause fatigue, somnolence, agitation, and aggression.^6^ Approximately 25% of patients fail to respond to either class,^7^ highlighting the need for novel, well-tolerated, and mechanistically distinct treatment options.

Drug repurposing can accelerate drug development and reduce translational barriers.^8^ In this context, there has been a growing interest in the role of l-type calcium channels (LTCCs), which modulate neuronal excitability, synaptic plasticity, and catecholaminergic transmission.^9^, ^10^, ^11^ LTCCs, including the Cav1.2 (*CACNA1C*) and Cav1.3 (*CACNA1D*) subtypes, are widely expressed in cortico-striatal and limbic circuits implicated in ADHD.^12^, ^13^, ^14^ Amlodipine (AML), an LTCC blocker, has been considered as peripherally acting with limited central nervous system (CNS) activity.^15^, ^16^, ^17^ However, emerging data imply greater blood-brain barrier (BBB) penetration than previously assumed and effects on behavioral domains relevant to ADHD, including hyperactivity and impulsivity.^18^^,^^19^

This review evaluates the potential for repurposing AML as a novel nonstimulant ADHD treatment. We begin by outlining the neurobiology of LTCCs in relation to attention and arousal, before examining AML’s pharmacology, CNS penetrance, and its emerging behavioral effects in both animal models and human studies. We conclude by outlining future research priorities and the broader implications for LTCC modulation in neuropsychiatric therapeutics.

## 2 l-Type calcium channels and ADHD: Biological plausibility

Evaluating AML’s therapeutic potential in ADHD requires placing it within the neurobiology of LTCCs. These channels regulate calcium influx into excitable cells, shaping processes relevant to ADHD pathophysiology.^9^, ^10^, ^11^ The 2 major pore-forming α1 subunits, Cav1.2 and Cav1.3, are widely expressed in brain regions implicated in attention and executive control.^12^, ^13^, ^14^ LTCCs also modulate monoaminergic signaling, with Cav1.3 supporting tonic firing and catecholamine output, Cav1.2 contributing to serotonergic and glutamatergic transmission, and both subtypes influencing burst firing, providing monoamine-dependent modulation of motor behavior, and supporting presynaptic dopamine (DA) release.^11^^,^^20^^,^^21^

Human genetics further support LTCC involvement. Genome-wide association studies implicate *CACNA1C* and *CACNA1D* as risk gene variants associated with several psychiatric conditions,^9^ including ADHD.^22^ Variants in the auxiliary subunits (eg, *CACNB1* and *CACNA2D3*), which modulate Cav1.2/Cav1.3 trafficking and function,^23^^,^^24^ have also been associated with ADHD.^19^ Interestingly, *CACNA1C*, *CACNA1D*, *CACNB1*, and *CACNA2D3* are direct targets of AML.^25^

In the ventral tegmental area, activation of Cav1.2 and Cav1.3 promotes burst firing in DA neurons, whereas Cav1.3 deletion slows tonic firing.^20^ Repeated LTCC activation in the ventral tegmental area reproduces behavioral sensitization,^26^ whereas LTCC blockade reduces conditioned place preference and relapse-like reinstatement induced by cocaine.^27^^,^^28^ Mechanistically, intracellular calcium inhibits potassium channels that support D2-autoreceptor negative feedback,^29^^,^^30^ providing a route by which LTCC dysregulation could destabilize DA signaling in circuits governing attention, reward, and behavioral inhibition. LTCCs also influence norepinephrine (NE) release, particularly from the locus coeruleus (LC),^31^ which innervates the prefrontal cortex (PFC) and supports alertness and working memory.^32^^,^^33^ However, excessive NE can impair PFC function.^34^^,^^35^ This is relevant to ADHD, in which PFC deficits have been reported.^36^

Beyond neuromodulation, LTCCs contribute to synaptic plasticity through long-term potentiation and depression,^37^^,^^38^ core mechanisms for learning and memory.^39^ Dysregulated intracellular calcium homeostasis and calcium-dependent signaling have been linked to altered connectivity and plasticity in ADHD,^40^^,^^41^ whereas LTCC blockade with AML improves working memory and executive function.^42^, ^43^, ^44^ LTCCs also regulate microglial activation and neuroinflammatory signaling,^45^^,^^46^ factors associated with ADHD.^47^

Collectively, these findings position LTCCs at an intersection of DA homeostasis, NE control, cortical plasticity, and neuroimmune regulation. This echoes a proposal made by Stanford^21^ that Cav1.2/Cav1.3 channels shape monoaminergic responses to stress and help govern a continuum from psychomotor impairment to hyperactivity and impulsivity, directly linking LTCCs to ADHD-relevant response control. AML, an LTCC inhibitor of Cav1.2/Cav1.3, is mechanistically positioned to address core neural features of ADHD.

## 3 AML’s mechanism of action: A systems-level view

We propose that AML can improve ADHD symptoms by modulating central LTCCs and downstream networks. In this review, we outline a systems-level model through which LTCC inhibition could translate into therapeutic benefit.

### 3.1 Synaptic plasticity and cortical signal integration

In hippocampal neurons, LTCCs regulate cyclic AMP-responsive element binding protein (CREB) phosphorylation and CREB-dependent gene expression that support long-term memory formation.^37^ LTCCs also modulate expression of brain-derived neurotrophic factor (BDNF), which mediates LTP in the thalamoamygdala pathway.^9^ Critically, the relationship between LTCC-mediated calcium influx and these plasticity mechanisms is not linear. CREB phosphorylation and BDNF transcription depend on tightly regulated calcium signals, where both the route of calcium entry and the amplitude and duration of the intracellular calcium transient determine the downstream transcriptional response.^48^, ^49^, ^50^ Excessive or sustained calcium influx through LTCCs can shift the balance from plasticity-promoting signaling toward excitotoxic or maladaptive cascades.^51^^,^^52^ This suggests an inverted U relationship in which both insufficient and excessive LTCC activity impair the fidelity of calcium-dependent plasticity.

This is directly relevant to ADHD in which working memory and executive function deficits are among the most consistently reported cognitive symptoms,^53^ and dysregulated calcium homeostasis has been linked to altered connectivity and plasticity.^40^^,^^51^ If LTCC overactivity in prefrontal circuits drives calcium transients beyond the range that supports efficient CREB and BDNF signaling, the result would be impaired synaptic strengthening and weakened cortical signal integration, consistent with the cognitive profile of ADHD. In animal models, AML-mediated LTCC blockade was associated with improved learning and memory performance.^42^, ^43^, ^44^ Rather than suppressing plasticity, partial LTCC blockade may restore calcium dynamics to a range that supports efficient CREB-dependent phosphorylation and BDNF expression, thereby improving working memory and executive function.

### 3.2 Modulation of dopaminergic tone and autoreceptor function

We hypothesize that central LTCC blockade may stabilize DA dynamics in ADHD (see section Reconciling LTCC blockade with dopaminergic models of ADHD). Excessive or unstable LTCC activity may promote erratic tonic firing of midbrain DA neurons, yielding inefficient signaling.^11^^,^^20^^,^^54^^,^^55^ By modifying LTCC-dependent pacemaker activity, AML may support more effective tonic firing and phasic DA responses, with potential benefits for attention, motivation, and impulse control. This is particularly relevant because ADHD has been characterized, at least in a subset of individuals, by attenuated tonic DA release alongside exaggerated phasic responses,^56^ suggesting that interventions acting on the biophysical properties governing firing patters, rather than directly augmenting or depleting DA, could selectively normalize signaling dynamics without producing the ceiling or floor effects associated with stimulant or reuptake-based pharmacology.

AML may further support dopaminergic control by improving D2-autoreceptor feedback. D2 autoreceptors provide negative feedback that limits DA release,^57^ but excessive LTCC-mediated calcium influx can desensitize these autoreceptors and weaken their regulation.^30^^,^^58^ By reducing intracellular calcium, AML could counteract this desensitization and improve dynamic control of DA release. Together, these 2 mechanisms—stabilization of firing patterns and restoration of autoreceptor sensitivity—represent complementary routes by which LTCC blockade could rebalance DA signaling in ADHD, addressing both the tonic deficit and the phasic excess that characterize the disorder.

### 3.3 Noradrenergic modulation

LTCCs shape noradrenergic signaling in part by regulating LC excitability,^31^ which determines NE tone in projections to the PFC.^33^ LC pacemaking arises from coordinated interactions among voltage-gated conductance, including LTCCs, and pharmacologic LTCC blockade can attenuate LC firing.^59^^,^^60^ NE levels exert a significant impact on PFC-dependent cognition. Although moderate NE supports working memory and top-down control,^61^^,^^62^ excessive NE recruits lower-affinity signaling and impairs PFC function,^34^^,^^35^ leading to reduced cognitive control.^63^, ^64^, ^65^ If ADHD-relevant PFC dysfunction involves LC hyperactivity or state-dependent NE excess, AML could decrease excessive LC-driven NE output and help maintain NE within a performance-optimizing range.

### 3.4 Dampening neuroinflammation

Excess intracellular calcium in glia can promote microglial/astrocytic activation and cytokine release,^66^ disrupting synaptic transmission, glutamatergic signaling, and monoamine synthesis.^67^, ^68^, ^69^, ^70^ Proinflammatory cytokines (eg, interleukins 1 and 6 and tumor necrosis factor α) can also activate indoleamine 2,3 dioxygenase, shifting tryptophan metabolism toward kynurenine products. These include kynurenic acid in astrocytes, inhibiting DA and glutamate release, and quinolinic acid in microglia, inducing oxidative stress by increasing free radical production and disrupting normal glutamatergic neurotransmission.^68^ Given links between ADHD and altered DA/glutamate signaling, oxidative stress, and neuroinflammation,^71^, ^72^, ^73^ AML-mediated calcium stabilization could reduce microglial/astrocytic activation and promote a more neuroprotective environment.

### 3.5 A multimodal therapeutic hypothesis

**Fig. 1:**
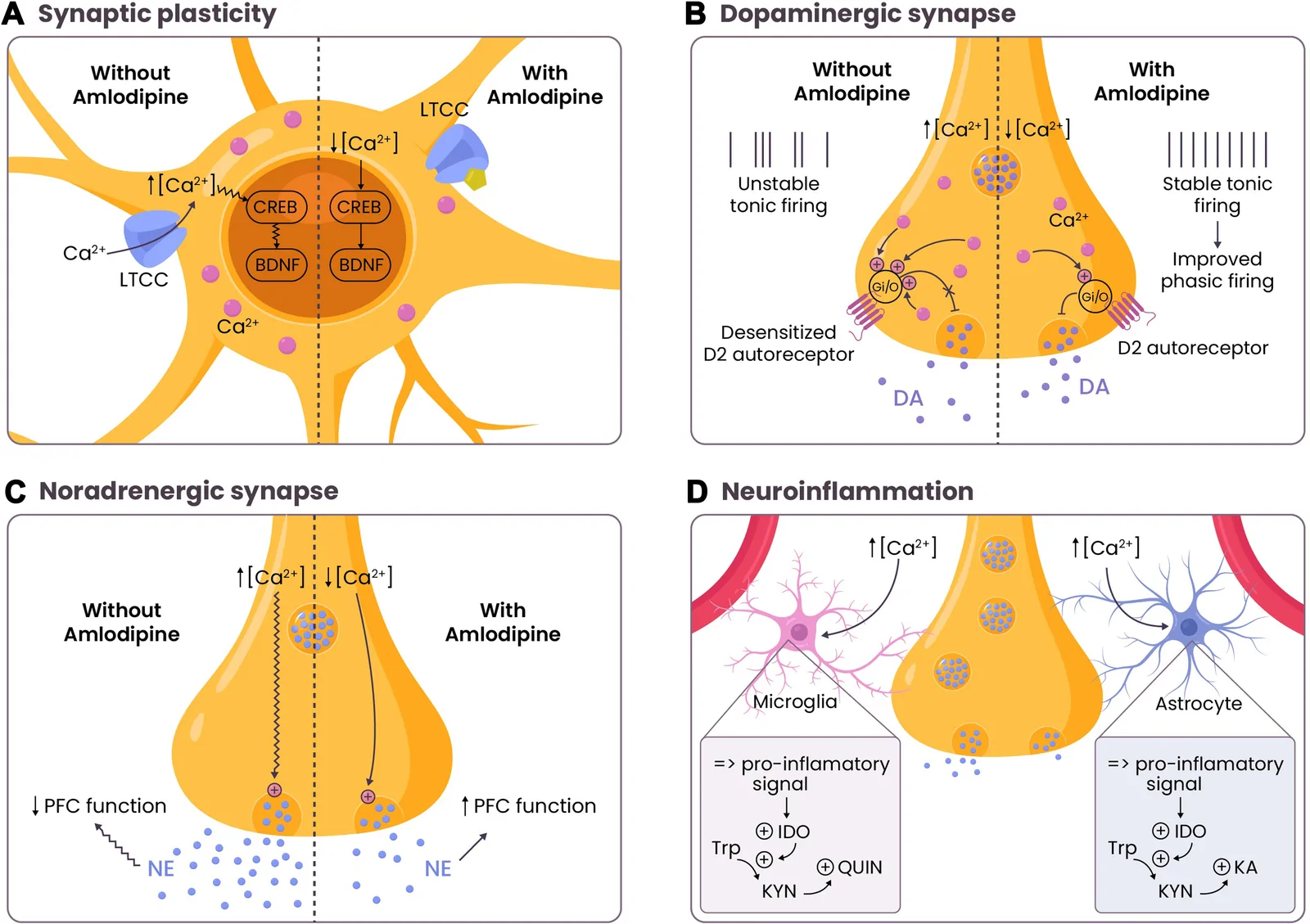
Overview of amlodipine’s mechanism of action in the central nervous system. Proposed cellular and neurochemical mechanisms by which amlodipine limits LTCC-mediated calcium entry and modulates synaptic plasticity, monoaminergic transmission, and neuroinflammatory signaling in an activity-dependent manner in a circuitry showing ADHD-relevant dysregulation. Serrated arrows denote uncontrolled signaling; straight arrows denote controlled signaling. (A) Synaptic plasticity. Without amlodipine, instability or overactivity of somatic LTCCs could lead to elevated intracellular calcium. Such dysregulation is poised to interfere with synaptic plasticity mechanisms, such as CREB-dependent transcription and BDNF expression, potentially affecting them negatively. Treatment with amlodipine reduces LTCC activity and dampens calcium influx, which in turn might help normalize CREB/BDNF signaling and support synaptic plasticity. (B) Dopaminergic synapse. In the untreated state, excessive LTCC activity may contribute to unstable tonic firing of DA neurons and desensitization of presynaptic D2 autoreceptors, potentially altering dopamine release. Amlodipine reduces LTCC-mediated calcium entry, which might stabilize tonic firing, improve phasic response patterns, and preserve D2-autoreceptor control of DA release. (C) Noradrenergic synapse. In an untreated state, excessive calcium influx could lead to impaired noradrenergic regulation, contributing to imbalanced PFC function. Amlodipine reduction of LTCCs activity could help stabilize NE release, which in turn might promote more balanced PFC function and neuronal homeostasis. (D) Neuroinflammation. Elevated calcium levels contribute to neuroinflammation by activating astrocytes and microglia to release proinflammatory cytokines. These cytokines stimulate IDO and shift Trp metabolism toward the KYN pathway, leading to increased QUIN production in microglia and KA synthesis in astrocytes. This increases oxidative stress and disrupts glutamatergic neurotransmission. Treatment with amlodipine might attenuate neuroinflammatory processes by lowering calcium levels and thereby reducing the inflammatory state. IDO, indoleamine 2,3-dioxygenase; KA, kynurenic acid; KYN, kynurenine; QUIN, quinolinic acid.

Integrating these pathways (Fig. 1), LTCC inhibition could (1) support synaptic plasticity and network integration (Fig. 1A), (2) rebalance tonic/phasic DA signaling and strengthen D2-autoreceptor feedback (Fig. 1B), (3) optimize NE output (Fig. 1C), and (4) attenuate neuroinflammation and oxidative stress (Fig. 1D). If validated, this multimodal profile could support a nonstimulant strategy, potentially as monotherapy or as an adjunct to existing treatments.

## 4 Crossing the BBB: AML’s CNS activity

The above-outlined mechanistic hypotheses depend on whether AML reaches the CNS at pharmacologically relevant concentrations. Therefore, we reassessed the longstanding claim that AML is peripherally restricted. This likely originates from Uchida et al^74^ and has been repeated in research for decades.^15^, ^16^, ^17^ Uchida et al^74^ measured brain entry of tritium-labeled AML in mice, reporting lower brain-to-plasma ratios than other LTCC blockers and negligible receptor binding in the brain, which was interpreted as evidence of limited CNS access. However, these findings have not been widely corroborated, and their interpretation is limited by methodological differences compared with current noninvasive approaches. Recent reviews note that invasive techniques, such as postmortem brain homogenization and radioligand binding, may not accurately reflect real-time drug dynamics in the living brain.^75^^,^^76^

AML’s effects are more difficult to interpret because its chemical properties differ from those of other dihydropyridines. Compared with other dihydropyridines, which are generally more lipophilic (logP ≈ 3.0–5.6),^77^ AML is near the lower bound (logP ≈ 2.96).^78^ It is also a weak base (p*K*_a_ ≈ 8.6), resulting in high ionization at physiologic pH.^79^ Combined with its long half-life (∼30–50 hours),^80^ these characteristics may facilitate steady, low-level CNS accumulation over time. Measurements of the unbound brain-to-plasma partition coefficient (*K*_p,uu,brain_) across species now provide direct evidence for this. In zebrafish, measurable brain concentrations were detected at the lowest dose that rescued hyperactivity and impulsivity in *adgrl3.1* mutants.^19^ Parallel mouse experiments indicate CNS penetration with high *K*_p,uu,brain_ (K. Ægir Karlsson, H. Þorsteinsson, H. Sóley Sveinsdóttir, D. Þöll Halldórsdóttir, and B. Grzymala, unpublished data; manuscript in preparation), and independent replication in rats also demonstrated significant CNS penetration.^19^ Collectively, these data challenge the characterization of AML as peripherally restricted and support central exposure as a plausible explanation for observed behavioral effects.

### 4.1 Functional CNS cross-species effects

Functional evidence further supports central activity. In a zebrafish model of ocular oxidative stress, AML restored behavior and reduced neuronal damage and oxidative stress markers in CNS tissue.^81^ These histological and biochemical outcomes confirm that AML exerts direct protective effects within the brain, reinforcing its CNS activity even under pathological conditions.

In a craniopharyngioma model, AML reduced tumor growth by disrupting calcium-dependent signaling in midbrain and hypothalamic regions, demonstrating central LTCC-linked pharmacodynamic effects in vivo.^82^ This study not only confirmed BBB penetration but identified central LTCCs as pharmacodynamic targets in vivo. The finding is particularly significant because the therapeutic mechanism, suppression of neuronal calcium transients, aligns with our proposed mechanism of action in ADHD, albeit with different downstream consequences.

Kerkhofs et al^83^ investigated AML in aged hypertensive BPH/2J mice, a model of cerebrovascular dysfunction. Chronic treatment normalized systolic blood pressure and variability and limited memory impairment. AML also decreased the extent of the BBB damage, likely because of its blood pressure-lowering effects. Although it did not affect microglia at BBB leak sites, it attenuated neuroinflammation globally, indicated by decreased microglial activation markers and morphological changes.^83^ These findings, in the light of the current *K*_p,uu,brain_ data, could be reinterpreted to assume that AML exerts direct cerebroprotective effects beyond vascular regulation by directly acting on microglia cells.

In a pilocarpine-induced status epilepticus model, postseizure administration of AML reduced Cav1.2 overexpression and intracellular zinc accumulation, decreased oxidative stress, preserved neuronal viability, maintained BBB integrity, and suppressed astrocytic and microglial activation.^84^ These findings suggest that AML has a direct effect on LTCCs in the CNS.

### 4.2 Reframing the pharmacological profile

Overall, the available evidence suggests that AML’s CNS effects have been underestimated. Rather than a purely peripheral vasodilator, AML may be more accurately conceptualized as a CNS-active LTCC inhibitor, with downstream effects on neuronal excitability and inflammatory signaling. This strengthens the rationale for evaluating AML in ADHD and supports broader interest in LTCC modulation across psychiatric conditions linked to calcium dysregulation, such as bipolar disorder and schizophrenia.^85^^,^^86^ More broadly, these findings prompt reconsideration of LTCC blockers as a class in psychiatry and challenge traditional boundaries between cardiovascular and neurological pharmacology.

## 5 Preclinical evidence supporting AML for ADHD

Given the established biological plausibility of LTCC involvement in ADHD, the next question is whether LTCC inhibition by AML produces measurable improvements in ADHD-relevant behaviors. Recent preclinical studies provide evidence supporting this hypothesis.

### 5.1 Behavioral rescue in zebrafish and rodents

An *adgrl3.1*-mutant zebrafish model was developed that exhibits an ADHD-relevant behavioral phenotype, specifically hyperactivity and impulsivity. In an unbiased compound screen, AML emerged as a candidate for rescuing the phenotype,^18^ prompting a reevaluation of its CNS profile and therapeutic potential. In *adgrl3.1*-mutant zebrafish, AML produced a dose-dependent reduction in hyperactivity.^18^ In a subsequent study, 12 additional LTCC blockers were evaluated using the same assay. Five showed no significant effects on hyperactivity, whereas the remaining 7 were limited by toxicity at moderate-to-high doses or exhibited greater behavioral variability than AML.^19^ Therefore, among LTCC blockers tested, AML uniquely demonstrated efficacy, tolerability, and reliability.

The failure of other LTCC blockers to replicate AML’s profile suggests that LTCC affinity alone is insufficient and that each compound’s pharmacology plays a decisive role. Therefore, several factors may account for the different performance of the comparator compounds. First, many dihydropyridines have short half-lives and rapid onset-offset kinetics, which produce transient peaks in plasma concentration that can trigger reflex sympathetic activation and introduce systemic stress,^87^, ^88^, ^89^ confounding behavioral readouts. In contrast, AML’s pharmacokinetic profile, characterized by gradual onset, sustained receptor occupancy, and minimal plasma fluctuation (see section Crossing the BBB: AML’s CNS activity), may afford more stable central LTCC modulation over the course of behavioral testing. Second, the toxicity observed at moderate-to-high doses with several comparators likely reflects off-target activity. Dihydropyridines vary in their selectivity across calcium channel subtypes, and some compounds interact with N-type or T-type channels, cardiac ion channels, or other receptor systems at concentrations that approach their LTCC-blocking range.^88^^,^^90^ Such off-target effects may narrow the therapeutic window, producing toxicity before a behaviorally effective dose is reached. Third, CNS penetration varies across dihydropyridines. Although compounds such as nimodipine and isradipine are recognized as brain penetrant,^91^^,^^92^ their shorter durations of action^93^ may limit sustained central engagement, particularly under chronic dosing conditions relevant to ADHD. AML’s capacity for gradual CNS accumulation (see section Crossing the BBB: AML’s CNS activity) may produce more consistent central effects than structurally related compounds with different disposition profiles.

Consistent with this, AML reduced impulsive responses in a 5-choice serial reaction time task in *adgrl3.1*-mutant zebrafish and attenuated hyperactivity in spontaneously hypertensive rats.^19^ Together, these results align with proposals that LTCC modulation may be particularly relevant to predominantly hyperactive and impulsive symptom profiles.^21^

### 5.2 Metabolomic and lipidomic convergence

Metabolomic and lipidomic profiling was conducted to link behavioral rescue with molecular alterations, by comparing wild-type (WT) and *adgrl3.1*-mutant zebrafish treated with AML, atomoxetine, guanfacine, methylphenidate, or vehicle.^94^ In *adgrl3.*1 mutants, AML shifted several metabolic pathways toward WT profiles. These pathways included lysophosphatidylcholines and other phospholipids involved in inflammation and cell signaling,^95^^,^^96^ amino acids essential for neurotransmitter signaling and synthesis,^97^, ^98^, ^99^ and folate biosynthesis relevant to methylation and neurodevelopment.^100^ Critically, AML was the only treatment that restored folate biosynthesis to WT levels and produced the strongest downregulation of lysophosphatidylcholines among all compounds tested, consistent with the anti-neuroinflammatory properties proposed in section A multimodal therapeutic hypothesis. Notably, all treatments, including AML, methylphenidate, atomoxetine, and guanfacine, restored taurine and hypotaurine metabolism to WT levels. This is particularly relevant given taurine’s role in calcium homeostasis and neuronal excitability,^101^ and evidence from rat studies showing that taurine administration improves hyperactivity and reduces inflammatory cytokine levels.^102^ These metabolic effects were unexpected because AML’s primary mechanism is entirely distinct from the monoamine reuptake or adrenergic targets of established ADHD treatments. Yet, the convergence on the same core pathways suggests that behaviorally effective ADHD therapeutics may share downstream systemic effects regardless of their initial pharmacological target, providing pathway-level evidence that AML engages disease-relevant biology in the *adgrl3.1* model.

### 5.3 Population-level and genetic corroboration

To evaluate translational evidence, UK Biobank data were analyzed to investigate associations between AML use and ADHD-related behavioral proxies, while accounting for genetic liability using an ADHD polygenic risk score (PRS). To date, no prospective clinical studies have evaluated AML in patients with diagnosed ADHD. The analyses described further therefore represent an indirect, population-level approach to detecting a potential behavioral signal. Because ADHD symptoms were not directly assessed at baseline, self-report items such as mood variability and risk-taking were used as proxies. In models including the PRS as a covariate, individuals with higher ADHD genetic liability who reported AML use had lower odds of endorsing mood swings or risk-taking behavior. No similar association was observed with ramipril, an angiotensin-converting enzyme inhibitor, suggesting that the effect is unlikely to be attributable to a general antihypertensive mechanism.^19^

This evidence relies on observational data and therefore does not establish a causal relationship. AML use may imply underlying cardiometabolic comorbidity and related demographic factors. Consistent with this interpretation, PRS analyses associated ADHD genetic liability with diabetes, obesity, and mood or anxiety traits. Prospective study designs and stratified analyses are necessary to distinguish drug effects from confounding variables.

Independent population-level data provide convergent, although indirect, support. In a Danish register study of over 3.7 million individuals, Kessing et al^103^ found that continued use of AML was associated with reduced rates of depression, a condition that frequently co-occurs with ADHD and shares underlying catecholaminergic dysregulation. In a separate line of evidence, Feldman et al^104^ followed >15,000 hypertensive patients for 11 years and reported that AML treatment was associated with a decreased risk of dementia compared to non-calcium channel blocking antihypertensives. More recently, analysis of the National Alzheimer’s Coordinating Center database found that AML use in patients with mild cognitive impairment was associated with a 32% lower risk of incident dementia.^105^ Although none of these studies assessed ADHD directly, they collectively challenge the characterization of AML as peripherally restricted and support the hypothesis that it exerts clinically meaningful effects on brain function.

## 6 The case for *S*-AML

AML is commercially available as a racemate containing equal proportions of *S*-AML and *R*-AML. Repurposing AML for ADHD requires selecting an enantiomer because of their distinct pharmacological profiles.^106^ *S*-AML is the active stereoisomer with about 1000-fold greater affinity for LTCCs than *R*-AML.^107^ *S*-AML is therefore likely responsible for the LTCC effects relevant to ADHD.

### 6.1 Enantiomeric pharmacology: A clean CNS profile

These pharmacological differences may result in distinct risk-benefit profiles. *R*-AML has been linked to increased nitric oxide (NO) production through the kallikrein-kinin pathway,^108^ contributing to peripheral edema and oxidative stress.^107^^,^^109^ If this effect extends to the CNS, excess NO may form reactive nitrogen species,^110^ potentially leading to BBB disruption due to oxidative stress,^111^ and promote release of proinflammatory cytokines.^112^^,^^113^ NO signaling may also perturb catecholaminergic signaling by, for example, increasing mitochondrial oxidative stress in LC neurons, inhibiting function of DA and NE transporters, and suppressing extracellular DA in the hippocampus.^59^^,^^114^, ^115^, ^116^, ^117^ Since dysregulation of DA and NE is involved in ADHD,^118^^,^^119^ these potential effects of *R*-AML could be counterproductive. Supporting this, formulations containing only *S*-AML have shown a reduced incidence of peripheral edema compared to racemic AML, while preserving antihypertensive efficacy.^120^^,^^121^

### 6.2 S-AML for ADHD: A targeted neuropsychiatric intervention

*S*-AML offers a targeted approach by enabling LTCC modulation in the brain while minimizing the adverse effects of *R*-AML. Mechanistically, *R*-AML induces NO release through kallikrein-kinin and NOS-dependent pathways, which is consistent with stimulation of kinin production rather than LTCC blockade.^108^ Consequently, *R*-AML may counteract desired neuropsychiatric outcomes, while the effects of *S*-AML are consistent with the LTCC hypothesis. Although current pharmacological data support the use of *S*-AML, ADHD-specific evidence is still needed. Direct comparative studies of *S*-AML, racemic AML, and *R*-AML are necessary to confirm mechanistic and clinical differences in neuropsychiatric applications.

## 7 Why the therapeutic potential of AML has been overlooked

Two primary factors have likely obscured the recognition of AML’s therapeutic potential in ADHD. First, minimal clinical overlap exists between populations prescribed AML and those diagnosed with ADHD. AML is mainly prescribed to older adults with essential hypertension,^122^^,^^123^ whereas ADHD is most often diagnosed in children and young adults and remains underdiagnosed in adults.^124^ This demographic separation limits chances to detect behavioral benefits in routine practice. Second, AML exerts context-dependent effects. In normotensive individuals, baseline vascular tone and calcium channel activity are low, such that LTCC blockade produces minimal hemodynamic change,^125^ whereas pronounced effects emerge when vascular tone is elevated.^126^ By analogy, AML may have little impact on neurotypical neural circuits operating near physiological set points but could selectively normalize dysregulated calcium influx and excitability in neural states associated with ADHD.

### 7.1 Reconciling LTCC blockade with dopaminergic models of ADHD

Molecular imaging studies indicate that ADHD is characterized, at least in a subset of individuals, by reduced tonic DA release alongside exaggerated task-evoked phasic DA responses, implying an elevated phasic-to-tonic signaling ratio.^56^ This pattern supports a view of ADHD as a state-dependent catecholaminergic dysregulation, rather than a uniform DA deficit. Under such conditions, interventions that act on underlying neuronal biophysics, particularly excitability and calcium-dependent plasticity, would be expected to exert effects that depend on baseline neural state and task demands.

Converging reinforcement learning frameworks further suggest that DA signaling encodes more than a single scalar reward prediction error (RPE). In distributional reinforcement learning models, heterogeneity across DA neurons, often conceptualized as differential sensitivity to positive versus negative RPEs, supports a population code representing the distribution of possible outcomes rather than only the mean expected value.^127^^,^^128^ Within this framework, mechanistic models of ADHD propose that reduced tonic DA combined with exaggerated phasic responses increases the gain on RPE-driven learning, leading to unstable value estimates and more variable, exploratory choice behavior.^129^ Empirical studies using computational modeling of behavior broadly support reinforcement learning abnormalities in ADHD, although the direction and magnitude of altered feedback sensitivity vary across tasks and cohorts.^130^

We propose that LTCCs may provide a cellular substrate for this gain effect because they contribute to DA firing pattern regulation and couple spiking to intracellular calcium signals that gate synaptic plasticity. In midbrain DA neurons, LTCC activation can promote phasic firing, and dihydropyridine LTCC antagonists suppress rhythmic and phasic activity.^20^ Because calcium amplitude and timing are key determinants of plasticity induction in corticostriatal learning circuits,^131^ LTCC-dependent calcium entry is well positioned to modulate how strong RPE-linked activity drives synaptic change.

Partial LTCC blockade with *S*-AML may therefore reduce excessive phasic-associated calcium influx and constrain RPE-driven plasticity when phasic DA signals are disproportionately large relative to tonic levels. In distributional terms, this would be expected to reduce overweighting of high-salience outcomes and potentially dampen feedback-driven volatility, without requiring a primary increase in baseline DA availability. This hypothesis predicts the largest behavioral effects in individuals and tasks characterized by elevated phasic/tonic DA dynamics and high trial-to-trial learning volatility, with smaller effects in neurotypical conditions where RPE scaling is relatively balanced.

## 8 Future directions: translational roadmap for *S*-AML in ADHD

Moving beyond historical oversight, it becomes essential to systematically validate these hypotheses. Hence, we outline a focused preclinical roadmap designed to rigorously assess the enantiomer-specific mechanisms proposed.

### 8.1 Preclinical enantiomer-specific characterization

A critical first step is the direct measurement of *K*_p,uu,brain_ for *S*-AML and *R*-AML to establish whether the enantiomers achieve comparable unbound concentrations in the CNS. Demonstrating central exposure is necessary to interpret enantiomer differences in behavioral and molecular outcomes as mechanistic, rather than as consequences of unequal brain penetration. Under this framework, therapeutic effects attributable to *S*-AML, together with absent or opposing effects of *R*-AML, would support enantiomer-specific pharmacology as the driver of efficacy. With differential CNS exposure established, spatial and single-cell multiomics, including transcriptomic, proteomic, and metabolomic profiling, can then localize *S*-AML target engagement in ADHD-relevant regions (eg, midbrain and PFC) and identify pathways associated with potential liabilities of *R*-AML. These datasets will propose mechanistic biomarkers for efficacy and tolerability, strengthening the case for enantiomeric separation.

Complementing these molecular approaches, in vivo/ex vivo electrophysiology will test *S*-AML effects on neuronal excitability and synaptic integration in DA and prefrontal circuits, while whole-brain zebrafish calcium imaging will map network-level responses to *S*-AML versus *R*-AML. Together, these assays provide both targeted validation of the proposed mechanisms (see sections *Synaptic plasticity and cortical signal integration, Modulation of dopaminergic tone and autoreceptor function, Noradrenergic modulation, and Dampening neuroinflammation*) and discovery-driven mapping of unanticipated circuit-level effects, generating a comprehensive preclinical profile of each enantiomer before clinical translation.

### 8.2 Translational and clinical pathways

Because clinical use has been almost exclusively limited to racemic AML, retrospective analyses of electronic health records, prescription databases, and biobank-linked phenotypic data should compare matched subgroups with and without AML exposure (eg, individuals with ADHD, relevant comorbidities, or treatment resistance). Any detectable behavioral or cognitive improvement would be notable because co-exposure to *R*-AML may dilute or obscure effects attributable to *S*-AML. In parallel, a physiologically based pharmacokinetic (PBPK) model for *S*-AML, integrating absorption, distribution, metabolism, elimination, and *K*_p,uu,brain_, will simulate CNS exposures across species and dosing regimens. Linking simulated exposures to preclinical pharmacodynamic readouts (behavioral rescue, circuit measures, and molecular signatures from section preclinical enantiomer-specific characterization) would enable evidence-based human dose projections, critical for Investigational New Drug-enabling and first-in-human studies.

Together, retrospective signal detection and PBPK-guided dose optimization provide complementary routes from preclinical evidence to clinical testing. The former identifies whether a behavioral signal is already detectable under real-world conditions with racemic AML, while the latter defines the exposure targets needed to design prospective trials of *S*-AML with adequate CNS coverage. More broadly, this roadmap illustrates how mechanism-driven repurposing and state-dependent pharmacology can uncover neuropsychiatric treatments within the existing pharmacopoeia.

## 9 Conclusions

AML is supported by multiple lines of evidence as a promising nonstimulant candidate for ADHD. Behavioral rescue in zebrafish and rodent models, pharmacokinetic data showing high CNS exposure, and population-level associations in genetically at-risk individuals support a central, rather than only peripheral, mechanism of action. In a systems-level model where LTCC inhibition modulates catecholaminergic signaling, synaptic plasticity, and neuroinflammatory pathways, these findings challenge the prevailing view that AML lacks relevance for neuropsychiatric applications.

Within this framework, *S*-AML emerges as the most rational candidate for further development. Its high affinity for Cav1.2 and Cav1.3, along with the absence of *R*-AML’s NO and kinin signaling, suggests a more selective and potentially safer CNS profile than the racemate.

Enantiomer-specific *K*_p,uu,brain_ measurements, spatial and single-cell multiomics, circuit-level electrophysiology, and whole-brain calcium imaging are needed to quantify target engagement and network effects. Retrospective signal detection in real-world cohorts, combined with PBPK modeling, can inform dosing strategies and patient stratification. Early-phase clinical trials of *S*-AML in well-characterized ADHD populations are required to determine whether this mechanistically motivated repurposing strategy can deliver safe and effective treatment.

An unexpected finding in zebrafish has progressed to a credible clinical development candidate with a defined regulatory pathway, mechanistic rationale, and translational strategy. More broadly, because dysregulated calcium signaling is implicated in conditions that frequently co-occur with or share neurobiological features with ADHD, including bipolar disorder, schizophrenia, and anxiety disorders, the LTCC-centered framework presented here may inform therapeutic exploration beyond ADHD itself. This reinvents the pharmacological role of AML beyond cardiovascular disease.

## Conflict of interest

Karl Ægir Karlsson and Haraldur Þorsteinsson report a relationship with 3Z ehf, which includes employment, equity or stocks, and travel reimbursement. Karl Ægir Karlsson and Haraldur Þorsteinsson are inventors on patents/patent applications relating to the use of amlodipine for ADHD treatment that are assigned to 3Z ehf, including EP4284356A2 (European patent) and a pending US patent application. Bartosz Grzymala, Dagmar Þöll Halldórsdóttir, Kristín Þorfinnsdóttir, and Hildur Sóley Sveinsdóttir are 3Z ehf employees. Other authors declare that they have no known competing financial interests or personal relationships that could have appeared to influence the work reported in this article.
